# Paris saponin VII suppressed the growth of human cervical cancer Hela cells

**DOI:** 10.1186/2047-783X-19-41

**Published:** 2014-08-15

**Authors:** Wenjie Zhang, Dian Zhang, Xi Ma, Zhaoyang Liu, Fang Li, Dongna Wu

**Affiliations:** 1 Maternal and Child Health Hospital of Xi’an, Xi’an, Shaanxi 710003, People’s Republic of China; 2 Department of Pathogen Biology and Immunology, Xi’an Medical University, Xi’an, Shaanxi 710021, People’s Republic of China

**Keywords:** Paris saponin VII (PS VII), Cervical cancer, Proliferation, Apoptosis

## Abstract

**Background:**

Saponins of several herbs are known to induce apoptosis in many cancer cells. The present study aimed to investigate the growth inhibitory effect of Paris saponin VII (PS VII), a kind of steroidal saponins from Chonglou (Rhizoma Paridis Chonglou), on the human cervical cancer cell line Hela and the relative molecular mechanisms.

**Methods:**

Hela cells were exposed to different concentrations of PS VII (1 to 100 μM). Inhibition of cell proliferation was measured by 3-(4,5-dimethylthiazol-2-yl)-2,5-diphenyltetrazolium bromide (MTT) and 5-ethynyl-2′-deoxyuridine (EdU) assays. The amount of apoptotic cells was evaluated by flow cytometric analysis. And the protein level of cleaved caspase-3, cleaved caspase-9, Bax, and Bcl-2 was evaluated by Western blot.

**Results:**

The half maximal inhibitory concentration (IC_50_) value of PS VII for the growth inhibition of Hela cells was 2.62 ± 0.11 μM. PS VII increased the expression of caspase-3, caspase-9, and Bax while decreased that of Bcl-2, suggesting that PS VII may induce apoptosis through intrinsic apoptotic ways.

**Conclusions:**

These data indicate that PS VII has the potential for the treatment of cervical cancer.

## Background

Cervical cancer is the third most common type of cancer in women worldwide, and a leading cause of cancer-related mortality, with an estimated 275,000 deaths in 2008 [1]. Despite considerable advances in surgical techniques and neoadjuvant chemotherapy, the survival rate of cervical cancer is still not remarkably improved. Platinum-based anticancer agents, represented by cisplatin, have therapeutic properties for patients with cervical cancer; however, toxicities, including myelosuppression, leukopenia, ototoxicity, neurotoxicity, and nephrotoxicity [2,3], may limit their long-term use. Therefore, it is required to develop new drugs with a more specific effect and low toxicity.

Natural products have been shown to be excellent and reliable sources for the pharmaceutical development of anticancer drugs [4]. Chonglou (Rhizoma Paridis Chonglou) is the root of *Paris polyphylla*. Phytochemical and pharmacological studies suggest that Chonglou has a wide range of medicinal activities, including anticancer, immunoregulatory, and cardiovascular effects [5]. It has been applied in the treatment of malignant lymphomas, lung cancer, nasopharyngeal carcinoma, brain tumors, and digestive system carcinomas. Polyphyllin and extracts from Chonglou show good antitumor effects *in vitro*, through inducing apoptosis, affecting cell cycle distribution, inhibiting angiogenesis, and regulating the immune function [6,7].

In the current study, we investigated the mechanism underlying the cytotoxic effects of a kind of steroidal saponins from Chonglou (Rhizoma Paridis Chonglou), namely, Paris saponin VII (PS VII), and its antitumor properties on Hela cell lines. The results indicated that PS VII inhibited the growth of the cell effectively. It upregulated cleaved caspase-3, cleaved caspase-9, and Bax expression in Hela cells. These preclinical studies indicated that PS VII may have potentials in the treatment of cervical cancer.

## Methods

### Chemicals and reagents

**Figure 1:**
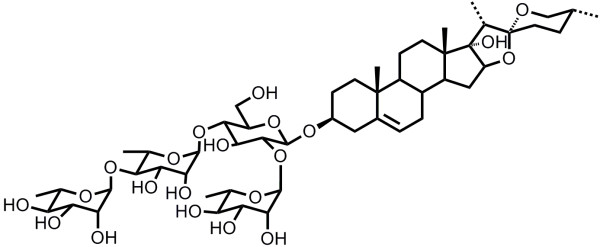
The structure of Paris saponin VII (PS VII).

3-(4,5-Dimethylthiazol-2-yl)-2,5-diphenyltetrazolium bromide (MTT), Hoechst 33342, and propidium iodide (PI) were purchased from Sigma (St Louis, MO, USA). Z-VAD-FMK was from Beyotime Institute of Biotechnology (Jiangsu, China). Anti-cleaved caspase-3, cleaved caspase-9, Bcl-2, and Bax antibodies were obtained from Cell Signaling (Beverly, MA, USA). Anti-β-actin antibodies were obtained from Santa Cruz Biotechnology (Santa Cruz, CA, USA). PS VII (Figure 1) with a purity of >98% was purchased from PureOne Biotechnology (Shanghai, China).

#### Cell culture

Hela, a human cervical cancer cell line, was obtained from the American Type Culture Collection (ATCC; Manassas, VA, USA) and maintained in DMEM medium supplemented with 10% fetal bovine serum, 100 U/ml penicillin, and 100 U/ml streptomycin. The cells were maintained in a humidified atmosphere at 37°C in 5% CO_2_.

#### Cell proliferation assays

Cell viability was determined by MTT and 5-ethynyl-2′-deoxyuridine (EdU) assays. The Hela cells were seeded at a density of 2 × 10^4^ cells/well in 96-well plates full of medium containing PS VII at various concentrations (1, 3, 10, 30, and 100 μM). After 24-h treatment, 20 μl of MTT solution (0.5 mg/ml) was added into each well and the mixture was incubated at 37°C for 4 h. The cells were then washed thrice with phosphate-buffered saline (PBS), and the formazan was resuspended in 150 μl DMSO. Absorbance was measured at 490 nm by using a Bio-Rad ELISA reader (Hercules, CA, USA). Half maximal inhibitory concentrations (IC_50_) of PS VII were determined by curve fitting analyses using Prism software (GraphPad Software, San Diego, CA, USA). For EdU assay, Hela cells (1 × 10^5^ cells/well) were seeded in 24-well plates. After culture in a serum-free medium for 24 h, various concentrations of PS VII (0.8, 1.6, and 2.4 μM) were added and the cells were kept for another 24 h. Cell viability was determined using an EdU assay kit. Stained sections were examined under a microscope (Nikon, Tokyo, Japan). All experiments were repeated independently thrice.

### Flow cytometric assays for annexin V

Hela cells were plated at a density of 5 × 10^6^ per 10-cm^2^ dish and cultured with different concentrations of PS VII for 24 h. A total of 1 × 10^6^ to 3 × 10^6^ cells were washed with ice-cold PBS and resuspended in 1× binding buffer [10 mM HEPES/NaOH (pH 7.4), 140 mM NaCl, 2.5 mM CaCl_2_] at a concentration of 1 × 10^6^ cells/ml. Five microliters of annexin V-fluorescein isothiocyanate (FITC) solution (25 μg/ml) and 5 μl of dissolved PI (250 μg/ml) were added to 100 μl of the cell suspensions. The cells were then gently vortexed and incubated at room temperature in the dark for 15 min. Then, 400 μl of ice-cold binding buffer was added and mixed gently before the cell preparations were examined by flow cytometry (FACSCalibur, Becton Dickinson, San Jose, CA, USA).

### Caspase-3 activity

Caspase-3 activity in Hela cells was detected using the Caspase-3 Activity Assay Kit (Beyotime Institute of Biotechnology, Jiangsu, China). The assay is based on the hydrolysis of the peptide substrate acetyl-Asp-Glu-Val-Asp *p*-nitroanilide (Ac-DEVD-pNA) by caspase-3, resulting in the release of a pNA moiety. Absorbance values were measured at 405 nm. Results were adjusted to the total protein content, and activity was expressed as nanomoles of pNA per milligram of total protein.

### Western blot analysis

Hela cells were incubated with 0.8, 1.6, or 2.4 μM of PS VII for 24 h. The cells were then harvested and resuspended in lysis buffer (Beyotime Institute of Biotechnology, Jiangsu, China, plus the protease inhibitors leupeptin 10 μg/ml, aprotinin 10 μg/ml, and PMSF 0.1 mmol/l). Protein lysates (30 μg) were electrophoresed on 15% SDS polyacrylamide gels and transferred to nitrocellulose membranes (Pall Corporation, Port Washington, NY, USA) and blocked with Tris-buffered saline (TBS) buffer containing 0.05% Tween-20 and 5% nonfat milk for 2 h at room temperature. The membranes were then incubated overnight at 4°C with various primary antibodies, followed by HRP-conjugated secondary antibodies. After washing the membranes thrice for 10 min in TBS buffer containing 0.05% Tween-20, the immunoreactive bands were detected using the Immobilon Western HRP Substrate (Millipore, Billerica, MA, USA). The experiment was repeated independently three times.

### Statistical analysis

Data were expressed as mean ± standard deviation (SD). Statistical analyses were done by using the one-way ANOVA test and Fisher’s least significant difference (LSD) *t* test to compare the different groups. Probability (*P* value) of less than 0.05 was considered to be statistically significant.

## Results

### PS VII inhibited the cell growth of Hela cells

**Figure 2:**
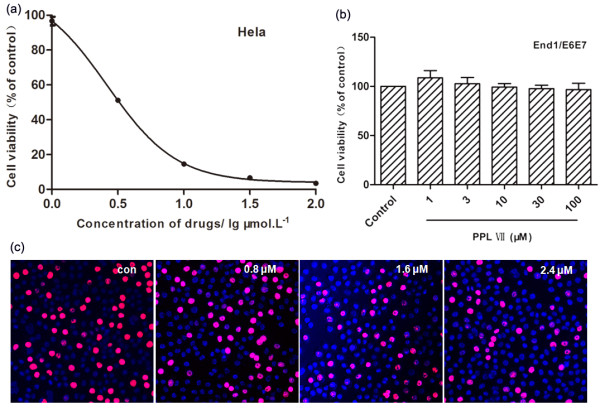
**The effect of PS VII treatment on the growth of Hela and End1/E6E7 cell lines. (a)** The effect of PS VII at the concentrations of 1, 3, 10, 30, and 100 μM on Hela cells. Common logarithm values of the concentrations were used as the *X*-axis. Lg1 = 0.0, Lg3 = 0.5, Lg10 = 1.0, Lg30 = 1.5, and Lg100 = 2.0. **(b)** The effect of PS VII at the concentrations of 1, 3, 10, 30, and 100 μM on the normal cervical epithelial cell line End1/E6E7. **(c)** The effect of PS VII at the concentrations of 0.8, 1.6, and 2.4 μM on Hela cells. Cell proliferation of Hela cells was evaluated by EdU assays. Red staining showed proliferative cells. Sections were counterstained with Hoechst (blue) to identify the orientation of nuclei (original magnification, ×200).

The viability of Hela cells treated with PS VII at different concentrations (1, 3, 10, 30, and 100 μM) for 24 h was determined by MTT assay. As shown in Figure 2a, PS VII decreased the cell viability rate in a concentration-dependent manner. In comparison to the control groups, treatment with PS VII at the concentrations of 3 and 10 μM decreased the survival rate of Hela cells to 51.16% ± 0.58% and 14.57% ± 0.24%, while at the concentrations of 50 and 100 μM, PS VII significantly (*P* < 0.01) reduced the viability rate of Hela cells to 6.63% ± 0.26% and 3.45% ± 0.13%. The IC_50_ value showed 2.62 ± 0.11 μM in Hela cells. To investigate whether this effect was selective, a normal cervical epithelial cell line, End1/E6E7, was treated with various concentrations (1, 3, 10, 30, and 100 μM) of PS VII for 24 h. The results show that PS VII had no obvious growth-inhibiting effect on End1/E6E7, even at the highest concentration of 100 μM (Figure 2b). Based on the IC_50_ value, in the following steps, PS VII at the concentrations of 0.8, 1.6, and 2.4 μM was chosen to treat Hela cells. Results of EdU assay (Figure 2c) also showed that PS VII exhibited an obvious growth-inhibiting effect on Hela cells.

### PS VII induced cell apoptosis in Hela cells

**Figure 3:**
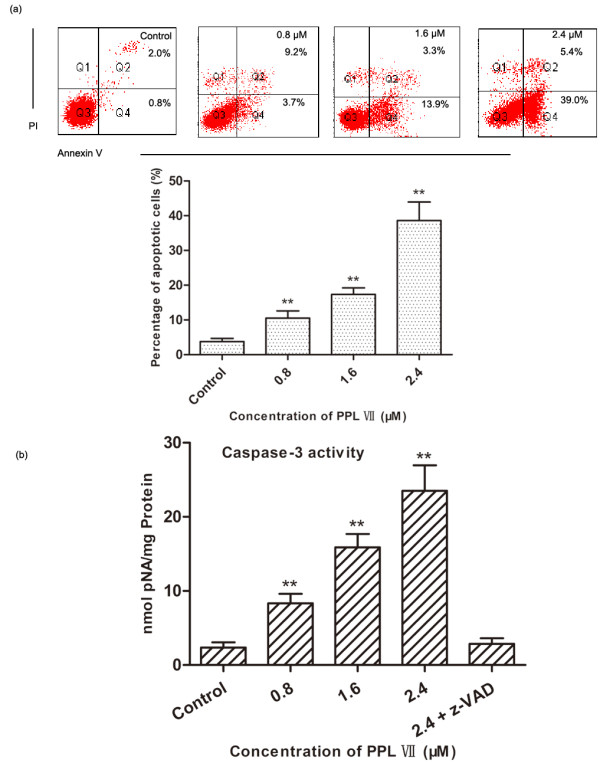
**The effect of PS VII treatment on the apoptosis of Hela cells. (a)** Annexin V analysis of Hela cells treated with PS VII at the concentrations of 0.8, 1.6, and 2.4 μM for 24 h. PS VII treatment increased the percentages of annexin V+/PI - (right lower quadrant) and annexin V+/PI + (right upper quadrant) cells. **(b)** Effect of PS VII on the caspase-3 activity of Hela cells. Data were the mean ± SD of three separate experiments. **P < 0.01 vs. control.

To investigate whether the growth inhibition of PS VII was caused by apoptosis, annexin V-FITC/PI assay was used. The percentage of apoptotic cells (Q2 + Q4) in Hela cells was 3.77% ± 0.91%. When exposed to 0.8, 1.6, and 2.4 μM of PS VII for 24 h, the percentage increased to 10.50% ± 2.12%, 17.37% ± 1.86%, and 38.60% ± 5.34%, respectively (Figure 3a). The activity of caspase-3 was also measured. As shown in Figure 3b, obvious activation of caspase-3 was observed in Hela cells treated with PS VII, which seemed to be in a concentration-dependent manner, whereas low caspase-3 activity was detected in the control group. The decrease activity of caspase-3 in the cells treated with PS VII was significantly blocked by pretreatment of a general caspase inhibitor, Z-VAD-FMK.

### PS VII acted on the intrinsic apoptotic pathway

**Figure 4:**
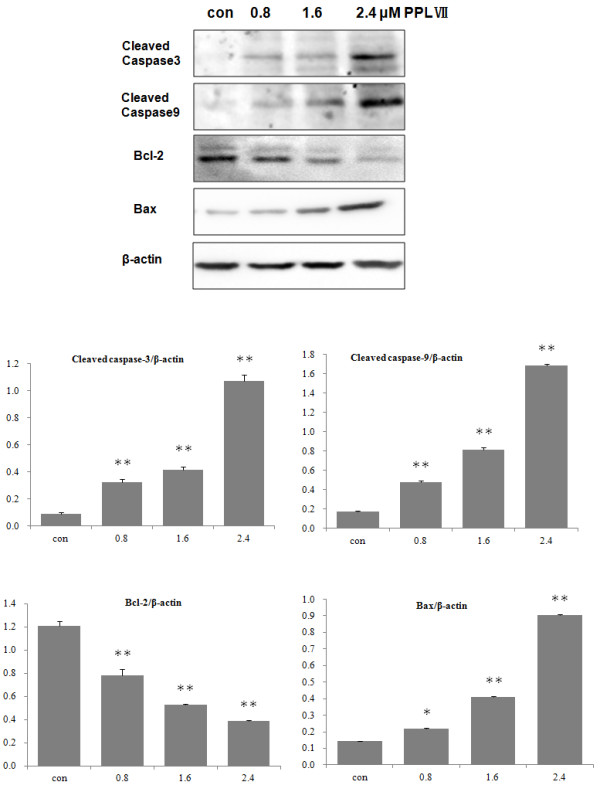
**The effect of PS VII treatment on apoptosis-related protein expression.** PS VII increased cleaved caspase-3, cleaved caspase-9, and Bax while decreased Bcl-2 expression in protein level in the Hela cells treated with 0.8, 1.6, or 2.4 μM PS VII for 24 h. ‘Quantity one’ of Bio-Rad and SPSS 17.0 were used to deal with the results of Western blot. *P < 0.05 vs. control; **P < 0.01 vs. control.

**Figure 5:**
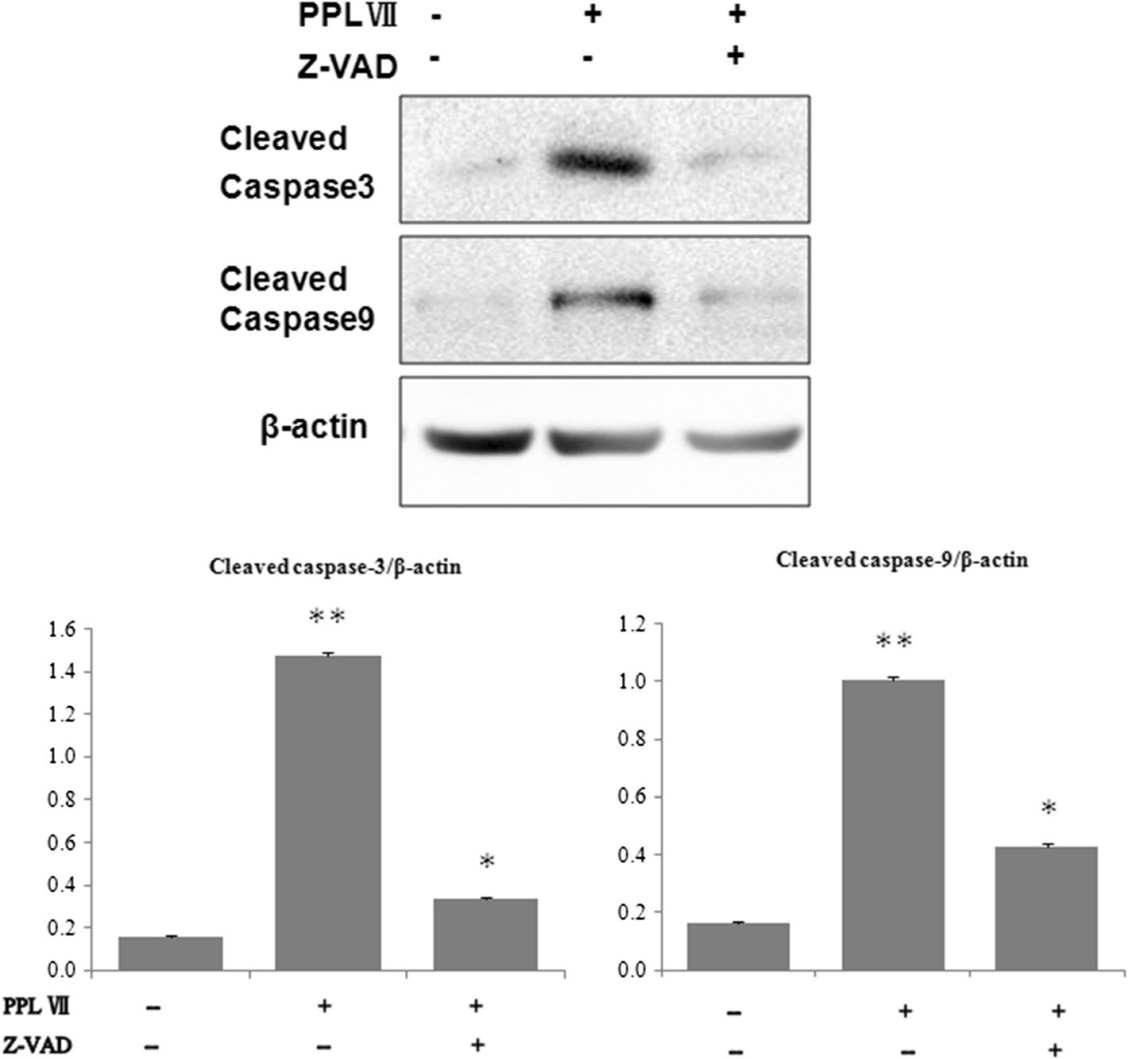
**Activation of caspase-3 and caspase-9 after treatment with PS VII.** Cell lysates were prepared after treatment with drugs for 24 h and then analyzed by Western blot with caspase-3 or caspase-9 antibody. The protein level of cleaved caspase-3 and caspase-9 was increased by PS VII treatment. However, the activation of caspase-3 and caspase-9 was minimized after pretreatment of a pan-caspase inhibitor (Z-VAD-FMK). ‘Quantity one’ of Bio-Rad and SPSS 17.0 were used to deal with the results of Western blot. *P < 0.05 vs. control; **P < 0.01 vs. control.

To search for the indication of mechanisms involved in apoptosis, the expression of cleaved caspase-3, caspase-8, and caspase-9 was evaluated. Compared with that in control cells, the expression of caspase-3 and caspase-9 significantly increased in Hela cells (Figure 4) while caspase-8 had no significant change (data not shown) after being treated with 0.8, 1.6, and 2.4 μM of PS VII for 24 h. In parallel to the alterations, Bcl-2 expression decreased while Bax expression increased. Furthermore, the decrease in the levels of caspase-3 and caspase-9 in the cells treated with PS VII was significantly blocked by pretreatment of a general caspase inhibitor, Z-VAD-FMK (Figure 5). These data suggested that PS VII induced cell apoptosis through an intrinsic pathway depending on caspase activation.

## Discussion

Chonglou (Rhizoma Paridis Chonglou) has been traditionally used for the treatment of microbial infection, hemorrhage, menometrorrhagia, and venomous poison [8-11]. The main bioactive ingredients of Chonglou are the steroidal saponins. Phytochemical and pharmacological studies have further revealed a novel therapeutic role as an anticancer agent for these steroid saponins [12]. However, evidence-based researches into the mechanism underlying the cytotoxic effects of steroidal saponins are still undefined.

In the current study, we found that PS VII, a kind of steroidal saponins from Chonglou, could inhibit the growth of Hela cells in a concentration-dependent manner. We then attempted to investigate whether the growth-inhibiting effect of PS VII was caused by apoptosis.

Apoptosis, which is characterized by membrane blebbing, shrinkage of the cytoplasm and nucleus, and DNA fragmentation [13], helps to keep tissue homeostasis by eliminating potentially deleterious cells. Deregulated apoptotic cell death would lead to diseases such as cancer. In cancer cells, the incidence of apoptosis and the rate of cell proliferation are uncontrolled, which would cause tumor invasion. Therefore, it will be a reasonable way to induce cancer cells to undergo apoptosis by various anticancer agents.

Results of flow cytometric analysis showed that PS VII increased the apoptosis of Hela cells in a concentration-dependent manner.

Mammalian cells undergo apoptosis mainly through two ways: the death receptor-mediated or extrinsic pathway and the mitochondrial-mediated or intrinsic pathway [14]. Caspases, a family of cysteine proteases, are central regulators of apoptosis. Caspase-8 is involved in the extrinsic and caspase-9 in the intrinsic pathway. After activation, they would cleave and activate downstream effectors such as caspase-3, which subsequently cleave cytoskeletal and nuclear proteins [15,16]. Results of caspase-3 activity assay showed that PS VII treatments led to increased caspase-3 activity in Hela cells. And these effects seemed to be concentration dependent. Western blot analysis demonstrated that PS VII treatments caused increased cleaved caspase-3 and caspase-9 expression, but had no significant effect on cleaved caspase-8 expression. The cleavage of caspases was prevented by pretreatment of Hela cells with a pan-caspase inhibitor, Z-VAD-FMK. These findings suggest that PS VII induced the apoptosis of Hela cells through an intrinsic way involving caspase activation.

This was proved by decreased Bcl-2 expression and increased Bax expression. The Bcl-2 protein family, which plays an important role in the intrinsic pathway, is divided into two functional subfamilies: pro-apoptotic proteins (Bax and Bid) and anti-apoptotic proteins (Bcl-2 and Bcl-xL). The ratio of Bax/Bcl-2 appears to be a critical determinant of a cell’s threshold for undergoing apoptosis. Results obtained above were from *in vitro* experiments. In the next step, we will assess the effect of PS VII *in vivo* and find the possible mechanisms by using xenograft models.

## Conclusions

In brief, the results reported herein demonstrated that PS VII inhibited the growth and induced the apoptosis of Hela cells effectively. One of the possible mechanisms involved is that PS VII triggered cell apoptosis via an intrinsic pathway depending on caspase activation. The precise signaling pathway remains to be further investigated. However, these data may provide an approach to treat cervical cancer.

## Competing interests

The authors declare that they have no competing interests.

## Authors’ contributions

WJZ and DZ participated in the design of the study and performed the statistical analysis. XM, DNW, and FL carried out the study and together with ZYL collected important background information. WJZ drafted the manuscript. All authors read and approved the final manuscript.
